# Harnessing sulfate-reducing bacteria with plants growing to revitalize metal-tainted coal mine soils in Midwest China: metal sequestration performance, ecological networking interaction, and functional enzymatic prediction

**DOI:** 10.3389/fmicb.2023.1306573

**Published:** 2023-11-15

**Authors:** Zhendong Yang, Qihong Wu, Zhenghua Liu, Xiang Qi, Zhenyu Zhang, Miao He, Cong Peng, Li Zeng, Jing Wang, Fan Li, Zhaoyue Yang, Huaqun Yin

**Affiliations:** ^1^School of Architecture and Civil Engineering, Chengdu University, Chengdu, China; ^2^Sichuan Provincial Engineering Research Center of City Solid Waste Energy and Building Materials Conversion and Utilization Technology, Chengdu University, Chengdu, China; ^3^School of Minerals Processing and Bioengineering, Central South University, Changsha, China; ^4^Infrastructure Management Department, Sichuan Academy of Medical Sciences and Sichuan Provincial People’s Hospital, Chengdu, China

**Keywords:** sulfate reducing bacteria, synergy, heavy metal sequestration, coal mining, microbial networking

## Abstract

Heavy metal contamination from coal mining calls for advanced bioremediation, i.e., using sulfate-reducing bacteria (SRB) technology. Yet, the interaction of SRB with native soil microbiota during metal sequestration, especially in the presence of plants, remains ambiguous. In this study, we assessed the metal sequestration capabilities, ecological network interactions, and enzymatic functions in soils treated with a predominant SRB consortium, mainly *Desulfovibrio* (14 OTUs, 42.15%) and *Desulfobulbus* (7 OTUs, 42.27%), alongside *Acacia dealbata* (AD) and *Pisum sativum* (PS) plants. The SRB consortium notably enhanced the immobilization of metals such as Zn, Cu, As, and Pb in soil, with the conversion of metals to residual forms rising from 23.47 to 75.98%. Plant inclusion introduced variability, potentially due to changes in root exudates under metal stress. While AD flourished, PS demonstrated significant enhancement in conjunction with SRB, despite initial challenges. Comprehensive microbial analyses revealed the pivotal role of SRB in influencing microbial networking, underpinning critical ecological links. This interplay between plants and SRB not only enhanced microbial diversity but also enriched soil nutrients. Further, enzymatic assessments, highlighting enzymes like *NADH:ubiquinone reductase* and *non-specific serine/threonine protein kinase*, reinforced contribution of SRB to energy metabolism and environmental resilience of the entire soil microbial community. Overall, this research underscores the potential of SRB-driven bioremediation in revitalizing soils affected by coal mining.

## 1. Introduction

Coal mining is the predominant mining activity in China, accounting for over 70% of the country’s total energy supported by 14 coal bases planned and constructed during the period of “Tenth Five-Year Plan” (Li, 2021). Indeed, the major coal production bases in China are predominantly situated in the Midwest provinces, including Shanxi, Shaanxi, Inner Mongolia, and Qinghai. This industry significantly impacts the environment and public health. The environmental degradation stemming from coal mining includes: direct effects such as excavation, substrate displacement, and waste disposal which lead to a decline in soil quality, rendering it less suitable for cultivation (Chattopadhyay and Chattopadhyay, 2020). Moreover, regions surrounding coal mines often exhibit elevated concentrations of metals. In certain areas, levels of heavy metals, such as lead and cadmium, have been observed to surpass safe thresholds by 3 to 4 times, resulting in pronounced heavy metal contamination (Liu et al., 2019). When these polluted soils are employed for agriculture, they introduce metals into the food chain and adversely affect crop yields. Populations residing near these mines display a marked incidence of heavy-metal-associated diseases (Kumari et al., 2023). Consequently, coal mines are prominent sources of soil metal contamination within the area.

As the ramifications of coal mine-associated soil metal pollution continue to draw scrutiny, a diverse array of remediation techniques have been championed, e.g., ion exchange, chemical precipitation, electrochemical treatment, adsorption, and bio-treatment (Song et al., 2022). These conventional methods, while having made significant strides in addressing contamination, are not without their limitations. Challenges often cited with these techniques encompass high operational costs, and the potential for producing secondary pollutants, which can be just as detrimental to the environment. Conversely, bioremediation stands out as a novel and compelling solution. Recognized for its eco-friendly nature, bioremediation employs biological agents, capitalizing on their natural processes to detoxify polluted soils. Its advantages, ranging from cost-effectiveness to high efficiency, have led to its growing endorsement within the scientific and environmental communities as a sustainable alternative to traditional remediation methods (Wang et al., 2021).

Sulfate-reducing bacteria (SRB) have emerged as an indispensable instrument in the bioremediation arsenal. Constituting a group of anaerobic microorganisms, SRB primarily reduce sulfate ions to generate hydrogen sulfide using organic compounds as electron donor. This characteristic mechanism serves as the linchpin in their ability to immobilize heavy metals. By converting these metals into their sulfide forms, SRB render them insoluble, effectively reducing their mobility and bioavailability in the environment (Zheng et al., 2021). This not only mitigates the direct toxic impact of metals but also aids in preventing their uptake by plants, thereby safeguarding the food chain. Numerous studies have validated the efficacy of SRB in heavy metal sequestrations. Zhang and Wang (2014) demonstrated that in the presence of ample carbon sources, SRB immobilized between 68.3 and 99.7% of metals including Fe, Mn, Ni, Zn, Cu, and Cd within a bioreactor setting. Qi et al. (2019) emphasized that besides conventional metal sulfide precipitation, the extracellular polymeric substances produced by SRB are rich in nonpolar and acidic amino acids, with the large negative charges in glutamic acid and aspartic acid specifically enhancing metal sequestration. Zhang et al. (2016) and Yan et al. (2018) indicated that, beyond just utilizing SRB, the introduction of carriers like graphene oxide serves to stabilize them, guarding against harsh conditions and potential inhibitors, resulting in an SRB growth rate of 0.27 h^−1^, a doubling time (td) of 2.5 h, and an impressive heavy metal precipitation efficiency of 97.1% from mine drainage. In terms of soil remediation, their success does not conclude with metal sequestration, whereas plants can be introduced to treated soils post SRB treatment. The now-immobilized metals pose a reduced threat, allowing for a more conducive environment for plants to thrive, further facilitating the soil restoration.

While the application of SRB to immobilize metals in aqueous environments is well-documented, their role in soil remediation and their interaction with indigenous soil microbiota and growing plants remains underexplored. Soil contains diverse bacterial communities that may compete or collaborate with SRB for energy and cellular resources before establishing stable coexistence. This complexity is accentuated when plants, with their root exudates and rhizospheric interactions, are introduced into the equation (Singh R. et al., 2018; Rahman et al., 2019; Adamczyk et al., 2021). Thus, critical questions arise for the effective application of SRB in soil remediation, particularly in the presence of plants: How does SRB network with the local bacteria, and how does this interaction affect the overall metabolic functions of the community? How does SRB remediation influence plant growth and the composition of its rhizospheric microbiota? Furthermore, metals interact with various natural ligands in soil, forming complex bonds that can influence the metal immobilizing functions of SRB, differing significantly from their behavior in water. The incorporation of plants adds an additional dimension of complexity, as root exudates might alter these metal–ligand interactions.

In light of the challenges of soils impacted by coal mining in China, the role of SRB in addressing heavy metal contamination is pivotal. Navigating the intricate dynamics of SRB within such unique soil environments, along with their interactions with local microbiota and plants, our study investigates the remediation capabilities of SRB in conjunction with two model plant species for soil restoration. We established varied conditions, either with or without SRB and plants, specifically *Acacia dealbata* (AD) and *Pisum sativum* (PS). Specifically, our objectives are: (i) To dissect the ecological relationship between SRB and the *in-situ* microbiota in coal mine impacted soils, focusing on community metabolic functions; (ii) To probe the interplay between SRB mediated soil remediation and plant growth; (iii) To delve into the nuances of metal binding in soil. Through this comprehensive exploration, we aim to foster a deeper understanding of SRB’s potential in remedying coal mine impacted soils, laying the groundwork for innovative, sustainable solutions to heavy metal contamination.

## 2. Materials and methods

### 2.1. Site description and soil sample collection

Soil samples were procured from a typical coal mining region in Midwest China. Specifically, samples were collected farmlands in Xiaomeidong Village, Qiaotou Town, Datong County, situated adjacent to the Datong coal mine in Xining, Qinghai Province of China (longitude 101^o^ 37′ to 101^o^ 56′, latitude 36^o^ 55′ to 36^o^ 58′, and an altitude of 2,450–2,750 m). This area, bordering the coal mining region, encompasses a total cultivable land of approximately 143 hectares with an average of 0.1 hectares per individual, with potential risks of soil heavy metal contamination. Sampling was conducted at 0–20 cm depths, using a core sampler with a 5 cm diameter and 60 cm depth at each site. Collected samples were dried at 40°C and subsequently sieved through a 2 mm mesh for further characterization and experiments.

### 2.2. SRB consortium isolation

Sewage sludge was used for SRB consortium isolation. Sewage sludge was procured from the anaerobic sector of Zhaotong City’s Second Wastewater Treatment Plant. This facility employs an oxidation ditch process, with a singular effective volume of 18,036.7 m^3^and an effective water depth of 5.6 m. The hydraulic retention time (HRT) is set at 22.43 h, divided among several zones: pre-anoxic (0.53 h), anaerobic (1.5 h), anoxic (3.6 h), and aerobic (16.8 h). The sludge age is maintained at 20 days. To cultivate the sulfate-reducing bacteria (SRB) consortium, a sequence of serial passaging was carried out using a modified sulfate broth medium containing: MgSO_4_ (2 g/L), Sodium citrate (5 g/L), CaSO_4_·2H2O (1 g/L), NH_4_Cl (1 g/L), K_2_HPO_4_ (0.5 g/L), Sodium lactate (3.5 g/L), and Yeast extract (1 g/L). The first passage commenced with 10 g of the sewage sludge added to 400 ml of this medium in sterilized anaerobic containers, while the futher passages include 100 ml of previous culture mixture with 300 fresh medium. Prior to the anaerobic stage, a 50 ml aliquot was reserved for analysis. The containers were then deoxygenated with nitrogen gas and incubated at room temperature for 1 week. After this period, a 100 ml cell suspension was extracted, with half subjected to analysis and the other half used for the succeeding passage in fresh medium. This procedure was reiterated five times, resulting in a refined SRB consortium designated for subsequent soil remediation experiments.

### 2.3. Soil remediation experimental setup

A series of controlled experiments were meticulously designed. Each experimental tray was uniformly loaded with 47.25 g of a designated soil sample, ensuring a standardized base for all setups. In the context of Group 1, a subset of trays was augmented with a 30% (w/w) concentration of the SRB consortium. A corresponding subset was equivalently supplemented with 30% (v/w) of a control medium. For Group 2, the focus was directed towards the *Acacia dealbata* (AD) as the chosen plant specimen. The soil treatments for this group paralleled those of Group 1 to ascertain the consistency and replicability of the observed outcomes. Conversely, Group 3 emphasized the *Pisum sativum* (PS) as the plant model. In alignment with the established protocol, one segment of trays for this group was integrated with a 30% (v/w) concentration of SRB consortium, whilst the counterpart segment was fortified with the 30% (v/w) control medium. To simulate natural growth conditions, all experimental setups were subjected to natural sunlight and irrigated thrice daily with a specified volume of 50 ml. Rigorous daily measurements were undertaken to document plant height, ensuring the mitigation of potential diurnal fluctuations. Upon the completion of the 15-day experimental period, a comprehensive analysis was conducted. This encompassed a detailed evaluation of soil physicochemical properties, microbial community composition, and phenotypic attributes of the plant specimens.

### 2.4. Analytical methods

Soil environmental properties were assessed. Elemental contents of C, N, and S were measured with a CHNS Elemental Analyzer EA1112 (Thermo Finnigan). Total solids (TS) were determined by drying soil at 50°C until a consistent weight was achieved. Volatile solids (VS) were found by heating the dried sample at 550°C for 6 h. The pH was measured potentiometrically after 24 h in a 1 M KCl solution with a 1/10 (m/v) ratio using a Mettler Toledo S400-B device. Bulk density was derived from the ratio of solid soil weight to its total volume. Soil porosity was based on its specific and bulk densities. Cation exchange capacity (CEC) was evaluated using a 0.1 N HCl extract, and Eh was gauged with an Eh Meter (SYS-OPR). Soil texture, including sand, silt, and clay, was determined through the hydrometer method as per Pansu and Gautheyrou (2006).

Soil metal composition was verified through X-ray fluorescence spectrum (XRF), using Canadian National Research Council’s Certified Reference Materials as a benchmark (Meija et al., 2010). Samples, combined with cellulose (4:1 ratio), were pressed into 25 mm × 4–5 mm pellets. A Canberra Si (Li) detector and ^238^Pu source were employed. Detection limits were calculated (Alvarez et al., 2007), factoring in a 6-h measurement time and standard deviation of the background window.

The metal species in soil were assessed using a sequential extraction protocol. Extracted metals were then analyzed with ICP-MS (iCAP RQ, Thermo Fisher Scientific) after mineralization. The oxidation–reduction potential (ORP) was gauged using a SYS-OPR portable meter. Sulfate content was determined via barium chromate spectrophotometry (Yang et al., 2023). AD and PS’s dry weight was established with an electric constant-temperature drying oven; after a 24-h drying period, the weight (post evaporation of cell water) of the plant parts was measured. Leaf count was done manually.

Total Cell density isolation was gauged through serial dilution in sterile broth and plating on Anaerobic Blood Agar. The most probable number assay estimated SRB density (Senthilmurugan et al., 2021). Biomass of the entire microbial community was determined using the Bradford protein assay (Bradford, 1976). Extracellular polymeric substances (EPS) extraction, as per Yue et al. (2015), utilized Ethylenediaminetetraacetic acid (EDTA). Subsequent measurements covered EPS’s total organic carbon (EPS-C) and its polysaccharide, protein, and nucleic acid content.

### 2.5. DNA sequencing and data mining

Total DNA was extracted using the SPINeasyTM DNA Kit (MP Biomedicals) post sediment homogenization. Amplification of primer pairs 341F and 805R occurred for 20 cycles (Yang et al., 2021). DNA and PCR product quality were assessed via a Nano Drop ND-1000 spectrophotometer (Thermo Fisher Scientific) and gel electrophoresis. Post multiple elutions, DNA was stored at −80°C, and a sequencing library was established using the Illumina Truseq Kit. 16S rRNA amplicons underwent sequencing on the Illumina Miseq platform. Raw sequence data processing utilized the QIIME platform (v 2020.6), refining sequences based on specified quality parameters (Caporaso et al., 2010). Sequences were categorized into operational taxonomic units (OTUs) at 97% similarity. RDP classifier allocated OTU classifications using the Greengene reference v13.8. A microbial interaction network was constructed based on OTU abundance correlations, adhering to set parameters. This network was then subdivided based on batch-specific OTUs. Network analysis utilized the “microeco” package v 0.11.0, with visualization in Gephi 0.9.1-beta (Liu et al., 2021). Microbial community function was estimated using PICRUSt 2, normalizing the OTU abundance table and comparing with the COG and KEGG libraries (Douglas et al., 2020).

## 3. Results and discussion

### 3.1. Physicochemical properties and heavy metals in coal mining-affected soil

To evaluate the impacts of coal mining on soil health and design remediation strategies, an analysis of soil physical and chemical properties was conducted, as illustrated in Table 1. The soil exhibited a substantial presence of carbon (116.13 ± 9.17 g/kg) and nitrogen (69.35 ± 5.38 g/kg), indicative of considerable organic matter content. However, given the coal mining context, a significant portion of this organic matter might be derived from coal residues, which are less favorable for soil fertility compared to plant residues (Weiler et al., 2020). The sulfur content of soil was measured at 14.36 ± 3.45 g/kg, suggesting that coal mining activities may be a contributing factor. This elevated sulfur level poses risks of soil acidification and nutrient imbalances. Additionally, the soil pH was determined to be 6.17 ± 0.86, indicating a slightly acidic environment. While this pH range is generally conducive to the growth of various plant species, it could exacerbate the risks of soil acidification, especially when considered in conjunction with the elevated sulfur levels and the possible influence of coal mining activities (Ma et al., 2020).

**Table 1:** Soil Properties including chemical composition and physical characteristics (mean ± standard deviation).

Parameters	Unit	Value
C	g/kg	116.13 ± 9.17
N	g/kg	69.35 ± 5.38
S	g/kg	14.36 ± 3.45
TS	%	35.87 ± 1.16
*VS*	%	74.05 ± 6.33
pH	NA	6.17 ± 0.86
Bulk density	g/cm^3^	0.62 ± 0.02
Soil porosity	%	37.16 ± 2.89
Sand	%	6.81 ± 0.24
Silt	%	58.37 ± 2.55
Clay	%	20.35 ± 2.13
CEC	Meq 100 g^−1^	9.67 ± 2.74

The cation exchange capacity (CEC) is 9.67 ± 2.74 Meq 100 g^−1^, indicating a reduced capacity for nutrient retention, which can adversely affect plant growth by limiting the availability of essential nutrients. The physical properties show a dominant silt content of 58.37 ± 2.55%, which can potentially lead to poor soil structure, prone to waterlogging and reduced aeration. The percentages of sand and clay are 6.81 ± 0.24% and 20.35 ± 2.13%, respectively, suggesting a texture that might be susceptible to erosion and compaction, further compromising plant root penetration and water infiltration (Helliwell et al., 2019). Despite these challenges, the soil maintains a low bulk density (0.62 ± 0.02 g/cm^3^) and a porosity of 37.16 ± 2.89%, implying favorable structure and water-holding capacity but with an underlying risk of waterlogging due to high silt content. These results generally present a soil environment impacted significantly by coal mining activities, exhibiting potential acidification and nutrient imbalances.

The XRF analysis was conducted to accurately assess the concentration of various metals in soil affected by coal mining (Table 2). The results revealed that Zn is the most prevalent, registering a concentration of 68.63 mg/kg. This finding aligns with previous studies that have reported high levels of Zn in similar environments due to its natural abundance in the earth’s crust and the release facilitated by mining operations (Li et al., 2017). Cu and As are also found in significant quantities, with concentrations of 21.5 mg/kg and 25.75 mg/kg, respectively. Pb is detected at a concentration of 16.83 mg/kg, a level that raises environmental concerns given its ability to be absorbed by plants, thus posing a threat to ecosystems and potentially infiltrating the food chain (Miller et al., 1975). Furthermore, Ni and Cd are present but in lower concentrations (1.57 mg/kg and 0.52 mg/kg, respectively). Hg, despite its known high toxicity and potential for bioaccumulation, which poses a risk to aquatic life and birds, is found at a low concentration of which is out of detection limit. This concentration, along with those of Ni and Cd, far more lower than the current standards on soil pollution, mitigating concerns regarding their immediate impact on the environment (Cepa, 2007). These results indicate a significant presence of metals, especially Zn, Cu, and As in the soil from coal mining areas, necessitating stringent monitoring and management strategies to mitigate environmental risks.

**Table 2:** XRF analysis results showcasing certified and measured metal concentrations, standard recovery percentages, and detection limits.

Metal	Certified con. (mg/kg)	Measured con. (mg/kg)	RR (%)	LD (mg/kg)
Hg	NA	0.033^*^	NA	8
Zn	70	68.63	98.0	5
Cu	23	21.5	93.5	3
As	27	25.75	93.4	2
Pb	18	16.83	93.5	4
Ni	1.9	1.57	82.6	1
Cd	0.7	0.52	74.3	5

con. Stands for concentration; RR stands for recovery rate; LD stands for detection limit; NA stands for not available.

* Suggests the value is unauthentic as it is out of LD.

### 3.2. Characterization and phylogenetic analysis of SRB consortium for soil remediation

To obtain an effective SRB consortium for further soil remediation, a serial passage method was employed, scrutinizing parameters including total cell density, SRB cell density, total protein, and extracellular polymeric substances (EPS) content across five passages (Table 3). The total cell density exhibited a marked increase, peaking at 23.2 × 10^7^ cell/ml in the fourth passage, and slightly receding thereafter, a trend indicative of a thriving microbial community nearing its optimal growth potential. Simultaneously, the SRB cell density amplified progressively, reaching a significant 13.7 × 10^7^ cell/ml in the final passage, showcasing the successful enrichment of the SRB consortium, a critical step towards achieving a potent consortium for remediation purposes. The total protein content, a marker for metabolic activity and biomass accumulation, surged initially, stabilizing around 71 mg/g in the latter stages, suggesting a high level of microbial activity and potentially reaching a balanced state of protein synthesis and degradation. The observed fluctuations in EPS content across the five passages appear to be part of a dynamic microbial ecosystem, influenced by a myriad of factors including nutrient availability and microbial interactions. Notably, EPS content reached a peak of 52.1 mg EPS/g TS during the third passage, subsequently declining in the fourth and fifth passages. One explanation for the peak in EPS content at the third passage could be linked to the increase in both total cell density and Sulfate-Reducing Bacteria (SRB) cell density. Elevated microbial activity may necessitate enhanced biofilm formation, a process largely mediated by EPS production, to facilitate cellular adhesion and nutrient acquisition (Ansari et al., 2017). The simultaneous increase in total protein content during this period further suggests heightened metabolic activity, which could drive higher EPS synthesis. The subsequent decrease in EPS content, despite a further increase in SRB cell density and a stable total protein level, may indicate a shift in microbial community behavior or environmental conditions. This could be due to nutrient limitations or changes in microbial interactions, potentially signaling a mature biofilm where EPS production is no longer as critical for community stability. Ultimately, the serial passage method has fostered a robust SRB consortium, evidenced by promising trends in cell density and protein content. However, the fluctuating EPS levels indicate a necessity for further optimization to achieve a stable and efficient microbial community for soil remediation.

**Table 3:** Serial measurements of total and SRB cell densities, total protein, and EPS values.

Serial No.	1	2	3	4	5
Total cell density (×10^7^ cell/ml)	1.7	12.5	18.5	23.2	19.7
SRB cell density (×10^7^ cell ml^−1^)	0.01	0.9	3.4	10.9	13.7
Total protein (mg/g)	3.9	48.7	69.3	71.5	70.7
EPS (mg EPS/g TS)	7.4	19.4	52.1	46.8	39.2

**Figure 1:**
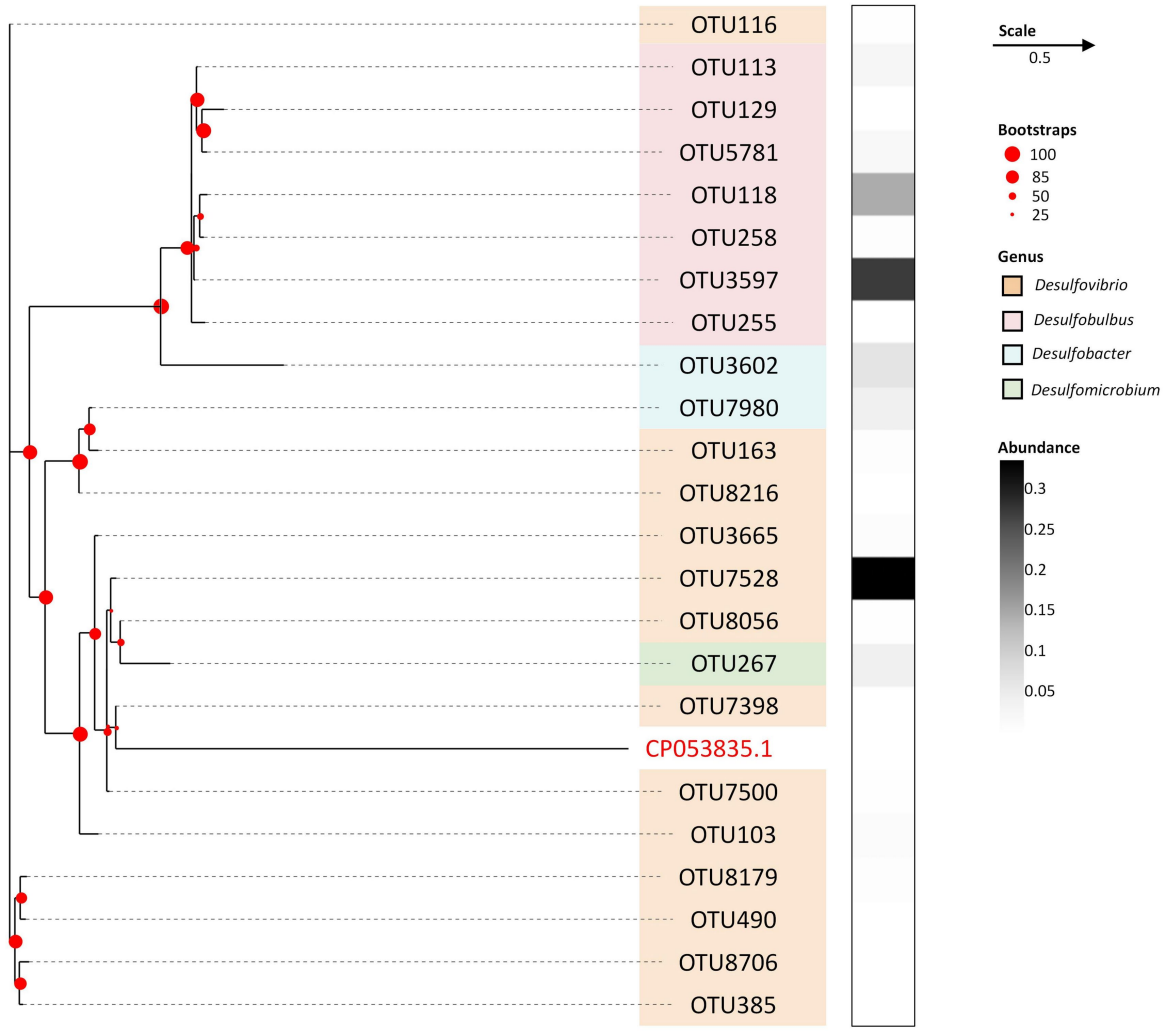
Phylogenetic tree of SRB at OTU level after serial passages. The orange grids refer to OTUs belonging to genus of *Desulfovibrio*, pink grids refer to OTUs belonging to genus of *Desulfobulbus*, blue grids refer to OTUs belonging to genus of *Desulfobacter*, Green grids refer to OTUs belonging to genus of *Desulfomicrobium*. The white to black grids refer to the relative abundance of each OTU against the total SRB community. The evolutionary history displayed on tree was inferred using the Neighbor-Joining method. The percentage of replicate trees in which the associated taxa clustered together in the bootstrap test (500 replicates) are shown with red dots on branches. A strain of *Arcobacter defluvii* was used for outsourcing, with an identifier of CP053835.1. in Gene bank.

To delineate the phylogenetic affiliations attributed to SRB within the screened consortium, a dendrogram was constructed based on hierarchical taxonomic classifications up to the genus level, as depicted in Figure 1. Remarkably, the dendrogram manifested a single pronounced cluster at a distance threshold of 5, encapsulating all the identified Operational Taxonomic Units (OTUs) and exhibiting a total distance range of 2.0 units. The consolidation of all OTUs into a singular cluster signifies a pronounced phylogenetic homogeneity, denoting a substantial overlap in taxonomic classifications among the SRB OTUs present in the consortium. This clustering pattern diverges from the typically observed microbial community structures characterized by a broader phylogenetic diversity, where OTUs are distributed across multiple clusters representing a variety of phylogenetic groups. In a comparative context, the conspicuous homogeneity observed in this study is notably distinct, potentially pointing to a specialized or narrowly defined ecological niche dominated by a specific phylogenetic group.

The OTU7528 emerges as the most abundant SRB, predominantly ascribed to the *Desulfovibrio* genus (Cabrera et al., 2006), constituting 33.55% of the total SRB abundance. It is closely followed by OTU3597 and OTU118, both attributed to the *Desulfobulbus* genus (Anandkumar et al., 2011), holding respective shares of 27.36 and 14.46%. Notably, while *Desulfovibrio* harbors the highest number of identified OTUs, totaling 14, its cumulative relative abundance of 42.15% is marginally surpassed by *Desulfobulbus*, which, despite having a smaller OTU count of 7, commands a slightly larger share of the overall SRB community at 42.27%. Such scenario implies a richer diversity in the *Desulfovibrio* population, albeit with a lesser collective abundance compared to *Desulfobulbus*. Conversely, genera such as *Desulfomicrobium* (Kushkevych, 2014) and *Desulfobacter* (Fang et al., 2019) were identified to a lesser extent, indicating their sparse representation in the consortium. This distribution underscores a competitive advantage for the *Desulfovibrio* and *Desulfobulbus* genera, facilitating their survival and nutrient competition in the sewage sludge origin, thereby delineating their significant ecological dominance in environments favorable to SRB. This observation aligns with findings from our previous studies, attesting to their adaptive superiority in such niches (Yang et al., 2023).

### 3.3. Soil rehabilitation dynamics: interplay of microbial and plant interactions

**Figure 2:**
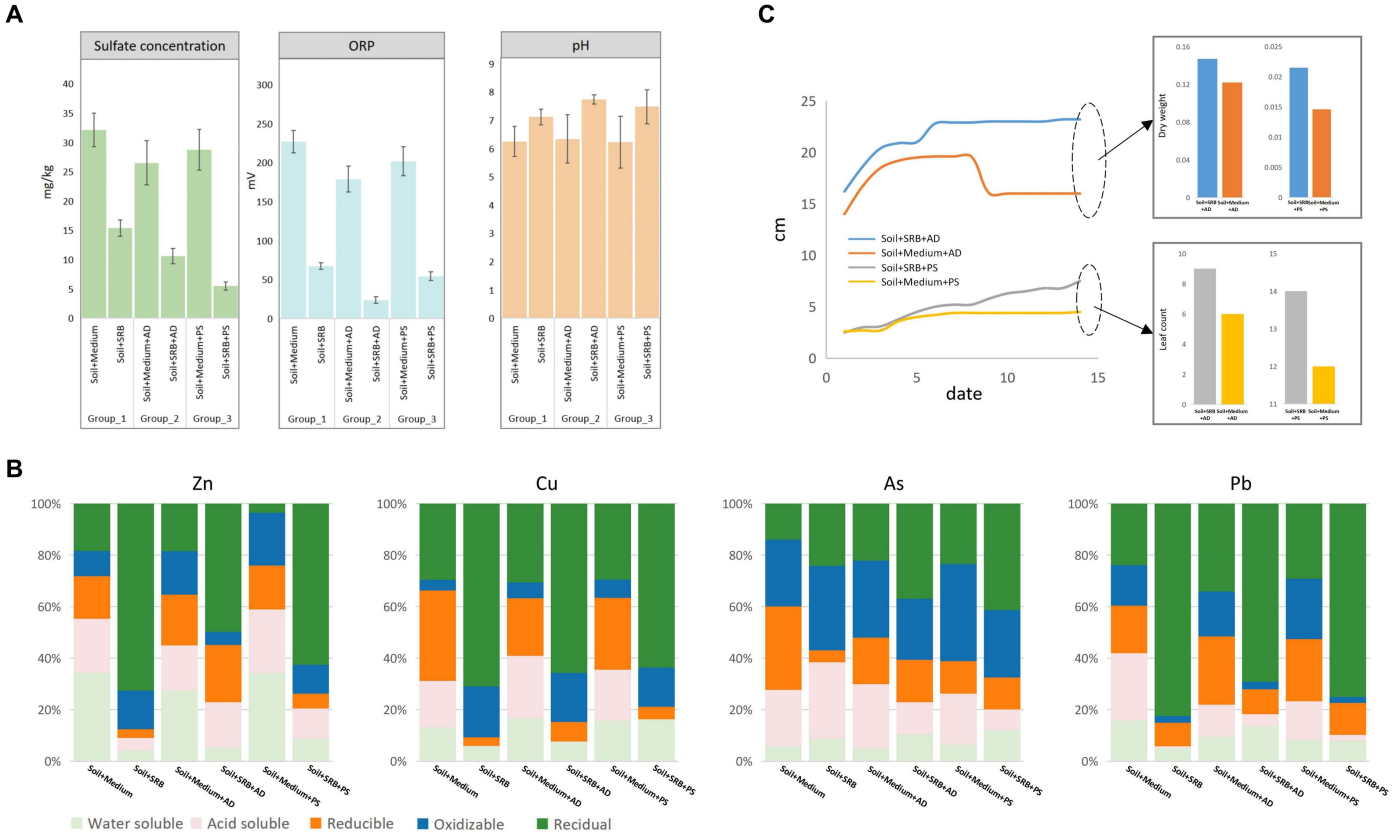
Comparative analysis of treatment performance towards soil across different setups. **(A)** Comparison of sulfate concentration, ORP, and pH levels. **(B)** Sequential extraction comparison of metals in soil highlighting distinctions in water-soluble, acid-soluble, reducible, oxidizable, and residual forms. **(C)** Growth analysis of AD and PS in soil, with and without SRB treatment, detailing both dry biomass weight and leaf count.

The SRB consortium was further supplied for soil remediation, complemented by phytoremediation using AD and PS. Figure 2A elucidates the intricate dynamics involving sulfate concentrations, ORP, and pH in soil affected by coal mining, portraying the transformative impact of various treatment strategies. The introduction of SRB significantly reduces sulfate levels from 32.14 ± 2.86 to 15.38 ± 1.36, and ORP from 226.85 ± 14.64 to 67.42 ± 4.04, while increasing the pH from 6.25 ± 0.53 to 7.12 ± 0.28, thus fostering an alkaline and reducing environment conducive to SRB activity. This marks the initiation of a healthy microbial ecosystem, laying the groundwork for future soil rehabilitation. Further analysis reveals that the incorporation of AD and PS influenced soil dynamics substantially, moderating sulfate concentrations in various setups, with PS enhancing sulfate management, reflected in values reaching as low as 10.62 ± 1.31. Moreover, a decline in ORP values is observed, with values dropping to as low as 23.62 ± 4.19 in a specific setup, illustrating the synergistic interplay between plant root exudates and microbial processes, thereby enhancing the potential to support reductive microbial activities (Singh V. K. et al., 2018). Additionally, the presence of both plant species is associated with a rise in soil pH, with levels elevating to as high as 7.74 ± 0.16, indicating a favorable modification in soil chemistry, potentially promoting nutrient availability and fostering a diverse microbial community (Sneha et al., 2021). This underscores the promising potential of leveraging plant-SRB synergies in rehabilitating coal mining-impacted soil, setting a stage for nuanced soil recovery strategies rooted in ecological principles. In the control (only with medium), plants exhibited minimal influence on sulfate reduction, ORP, and pH alterations. However, with the introduction of SRB, a marked decrease in sulfate concentration was observed, accompanied by significant shifts in ORP and pH.

Sequential extraction results, as depicted in Figure 2B, highlight the speciation and potential mobility of mainly identified metals (Zn, Cu, As, and Pb) in soil subjected to various treatments. The addition of SRB notably increases the residual form of all metals compared to the control soil with only post-age medium. Specifically, the water-soluble fraction of Zn is reduced from 35.25% in the control to 3.44% with SRB. However, the introduction of plants, AD and PS, seemed to counteract this immobilization. The residual fraction of Zn in soil with AD is 45.90%, lower than the 59.57% in SRB-only soil. For Cu, the water-soluble fraction is slightly reduced from 12.24% in the control to 5.42% with SRB, while the residual form sees a significant increase from 27.49% in the control to 65.39% with SRB. Furthermore, the acid-soluble fraction drops dramatically from 16.75% in the control to a mere 0.02% with SRB. When AD and PS are introduced, the oxidizable fraction increases, recording 17.53 and 16.27%, respectively, when combined with SRB. These results suggest that the interaction between SRB and plants might release metals. One potential mechanism could be the alteration of root exudates, which play a pivotal role in plant-microbe interactions, shaping microbial communities and mediating resource competition (Rahman et al., 2019). Additionally, plant hormones, when under heavy metal stress, can influence the bioavailability and mobility of metals, potentially affecting the speciation observed (Emamverdian et al., 2020). As presents a distinct pattern, with AD and PS, its immobilization is enhanced, with the oxidizable fraction in soil combined with SRB and AD at 23.66%, higher than the 4.64% in SRB-only soil. A significant portion of As is in the oxidizable form, potentially linked to As^3+^, a common species in coal mine-affected soil (Raj et al., 2022). Pb in soil with SRB shows a high residual form of 75.98%. However, with AD, this decreases to 63.68%, suggesting potential interactions between AD and SRB affecting metal speciation. While the presence of SRB accentuates metal sequestration, the introduction of plants introduces complexities, potentially due to alterations in root exudates and hormonal responses under metal stress.

**Figure 3:**
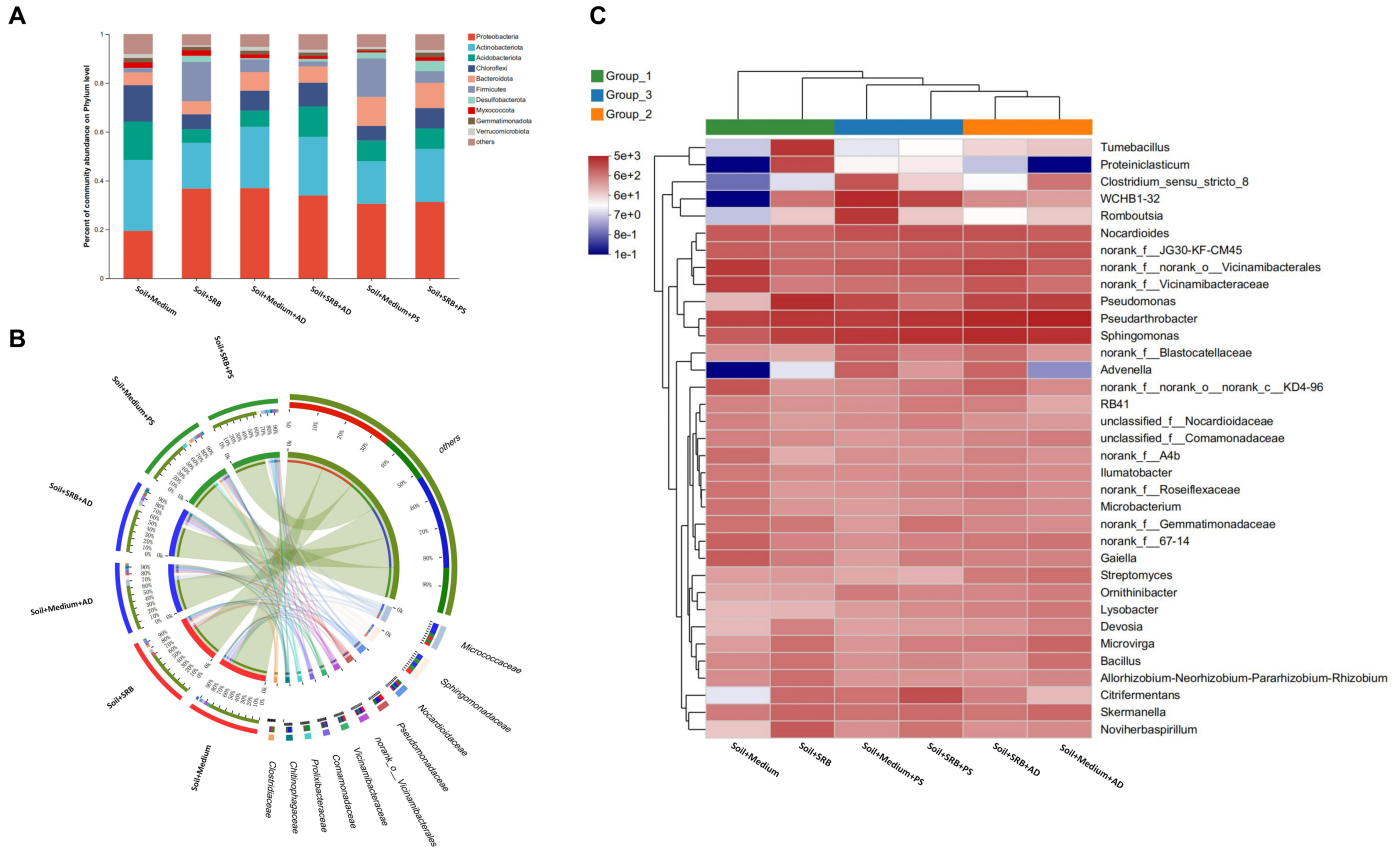
Microbial composition in soil across different setups. **(A)** Bar chart showcasing the top 10 most abundant phyla; **(B)** Circos plot illustrating the microbial composition at the family level; **(C)** Heatmap presenting the microbial composition at the genus level. The color code displays the z-score of each genus in the graph: the red scale represents data points above the mean, whereas the blue scale represents data points below the mean.

Figure 3C showcases the growth dynamics of AD and PS in soil with and without SRB as a function of time. In soil supplemented with SRB, AD exhibits a notable growth advantage, with its stem length reaching 23.2 cm by day 14, compared to 16 cm in the control soil. This is further corroborated by the dry weight measurements, where AD in the SRB supplemented soil accumulated 0.1471 g, surpassing the 0.1220 g observed in the control. For PS, the growth trajectory is more intricate. In the SRB supplemented soil, its stem length progressively increased to 7.5 cm by day 14. In contrast, in the control soil, its growth appears to plateau, stabilizing at 4.5 cm. This trend is consistent with the biomass data, where PS in the SRB soil achieves a dry weight of 0.0215 g, a marked improvement from the 0.0146 g in the control. Notably, in the absence of SRB, PS experiences mortality in the later stages, emphasizing the pivotal role of SRB in promoting PS growth in slag-contaminated soil. The enhanced growth observed in the presence of SRB can be attributed to several reasons. SRB might improve soil structure, making it more conducive for plant root penetration and water retention (Querejeta, 2017). Additionally, SRB can boost nutrient availability by stimulating the microbial sulfur cycle, which involves transforming elemental sulfur into carbon-bound sulfur essential for the production of amino acids (Etesami and Adl, 2020). Another reason, apparently, is that they reduces the metal toxicity by immobilizing them in the soil, thus diminishing the phytotoxic effects on plants (Bert et al., 2009). Collectively, the results underscore the beneficial role of SRB in augmenting plant growth. While AD demonstrated robust overall growth, PS, despite its lower absolute metrics, exhibited a significant relative growth advantage in the presence of SRB.

### 3.4. Microbial composition responses to SRB and plant interventions in coal mining-affected soil

SRB offer multifunctional benefits in the context of metal-contaminated soil. Not only do they directly reduce metal bioavailability, but they also potentially stimulate the growth and activity of indigenous microbiota. Such native microbial community plays a pivotal role in metal biotransformation and in fostering beneficial plant interactions essential for soil restoration. By amplifying the activity of these native microbes, SRB indirectly boost plant health and accelerate the soil restoration process. To elucidate the impact of SRB as well as their interaction with plants to local microbial community, microbial composition in soil across different setups is carried out (Figure 3). Prior to a focused exploration of microbial compositions, a preliminary assessment of microbial diversity is undertaken (data not shown). Distinctively, settings characterized by the presence of SRB and supplemented with AD displayed significant biodiversity. In contrast, conditions constituted solely of a basic medium substrate showcases reduced microbial diversity. This differential microbial landscape potentially underscores the influence of biotic factors, such as the presence of specific plants, on the microbial community structure.

Figure 3A illustrates the microbial community composition in soil with distinct treatment conditions, emphasizing the influence of SRB as well as the AD and PS. In soil absent of SRB, the phylum Proteobacteria is observed to constitute 19.5% of the microbial assemblage. Concurrently, Actinobacteriota manifests a substantial representation, accounting for 29.1% of the community. However, upon the incorporation of SRB, a marked shift is observed. Proteobacteria augments its dominance to 36.8%, while Actinobacteriota registers a relative abundance of 18.9%. Notably, Firmicutes exhibits a pronounced elevation from a baseline of 1.6 to 16.0% in SRB supplemented conditions, suggesting a potential ecological preference within such environments. Further delineation with the introduction of AD reveals nuanced microbial dynamics. Proteobacteria sustains its predominance, representing 37.0% in AD-influenced conditions. However, Actinobacteriota experiences a reduction to 25.2%. Remarkably, in environments juxtaposing SRB and AD, Proteobacteria constitutes 33.9%, whereas Firmicutes undergoes a substantial decline to 2.0%. Such observations underscore the intricate interplay and potential competitive/cooperative interactions within these microbial communities. Upon evaluation of PS influenced soil, distinct community structures are discerned. Bacteroidota emerged more prominently, accounting for 11.9% of the community. Meanwhile, Proteobacteria persists in its dominant role with a representation of 30.5%. In conditions amalgamating both SRB and PS, Proteobacteria maintains a substantial 31.2% presence, with Firmicutes exhibiting relative stability at 4.8%. Overall, the microbial community composition demonstrates significant variation. The dominance of Proteobacteria across diverse conditions underscores its pivotal ecological role. Moreover, the fluctuating abundances of Actinobacteriota and Firmicutes in response to SRB and plant introductions highlight the intricate balance and adaptability of microbial communities.

Figure 3B offers a granular perspective on microbial composition at the family level. In SRB supplemented soil with AD, the *Micrococcaceae* family from *Actinobacteria* stands out, known with its versatile organic matter decomposition capacities that increasing soil fertility (Macías-Benítez et al., 2020), with its abundance reaching a striking 8.7%. This prominence contrasts sharply with soil dependent solely on medium, where the presence of *Micrococcaceae* wanes to approximately 4.0%. Further, in soil augmented with SRB, the *Prolixibacteraceae* family emerges with high abundance, especially when PS is present, marking an abundance of around 6.9%. This flourishing contrasts with SRB deprived conditions, where its abundance takes a downturn. Known for their roles in specific soil nutrient cycles, such families delineate the profound influence of SRB and plant synergies (Barton and Fauque, 2022). *Pseudomonadaceae*, a family from Proteobacteria, displays a marked affinity for SRB-rich soil, registering an abundance peak of about 5.3%, irrespective of the vegetative overlay. *Pseudomonadaceae* are recognized for promoting the second phase of the electrogenic process under sulfate-reducing conditions (Pérez-Díaz et al., 2020). This trend suggests potential ecological niches that the family occupies, favoring SRB supplemented conditions. Drawing from these observations, while the overarching influence of SRB sets the microbial trajectory, specific families, modulated by interactions with AD and PS, navigate the nuanced ecological intricacies of these soil.

To elucidate the effects of SRB alone and in conjunction with plants on the soil microbiome, the microbial composition at the genus level was investigated (Figure 3C). *Pseudarthrobacter* emerges prominently, constituting 4.2% in untreated conditions and escalating to 5.1% under SRB treatment. Renowned for its role in carbon cycling, this representative genus from *Micrococcaceae* plays a crucial part in organic carbon turnover (Son et al., 2023), essential for restoring soil organic matter in mining-impacted areas. *Pseudomonas* witnesses a remarkable increase, from an initial 0.2 to 6.7% upon SRB introduction. This genus produces biosurfactants that boost plant nutrient uptake and inhibit fungal pathogens by disrupting their cell functions (Singh R. et al., 2018). *Sphingomonas*, starting at 2.1%, climbs to 6.9 and 5.9% in the presence of SRB and AD and PS, while only with SRB it also shows a relatively high abundance of 5.8%. Noted for its strong phosphorus utilization, especially phosphorus solubilization (Asaf et al., 2018), the increased presence suggests that AD releases substrates favored by this genus. Concurrently, *Sphingomonas* likely facilitates nutrient acquisition for the plant, addressing phosphorus deficiencies commonly found in degraded terrains. The *Vicinamibacterales*, a less known taxa in literature, registering at 4.9%, diminishes to 1.5% with SRB but rebounds to 4.1% alongside AD. In turn, such observation is not found in soil with SRB and PS. Moreover, unclassified genera, starting at 6.1%, peak at 6.0% with SRB and adjust to 5.8% with PS and SRB. Their fluctuation suggests unexplored microbial roles for soil restoration. Apparently, the combined presence of AD and SRB has a more pronounced impact on microbial composition compared to SRB alone, whereas the effect of PS with SRB is less distinct.

### 3.5. Molecular network analysis: deciphering SRB corresponding to microbial adaptability

Molecular ecological network analyses of each treatment is analyzed to grasp microbial adaptability changes. Network properties, as illustrated in Table 4, emphasize the structural nuances across various treatments. Treatment supplemented with only medium exhibits a distinct network structure, while another, augmented with SRB, displayed notable variations in properties like modularity. Upon introducing AD and PS, there is a noticeable shift in the clustering coefficient compared to the medium-alone soil. In fact, plant root micro-environments significantly influences network clustering of microbial constituents (Yuan et al., 2022). In terms of graph density, the environments with the microbial medium and those enhanced with SRB maintains a level of consistency. However, the inclusion of two different plant species shows distinct variations. Delving into average degree and path length, the soil integrating the SRB and AD/PS demonstrates obvious alterations in connectivity and network spread. In general, while a microbial medium dictates certain foundational network characteristics, the addition of SRB and specific plants introduces multifaceted patterns.

**Table 4:** Overall characteristics of molecular ecological networking in different setups, illustrating network diameter, modularity, clustering coefficient, graph density, average degree, and average path length values.

Sample ID	Network diameter	Modularity	Clustering coefficient	Graph density	Average degree	Average path length
Soil + Medium	10	0.26	0.65	0.04	5.02	2.74
Soil + SRB	8	0.53	0.67	0.04	6.01	2.22
Soil + Medium + AD	12	0.39	0.54	0.05	8.76	4.28
Soil + SRB + AD	7	0.57	0.60	0.05	9.83	2.83
Soil + Medium + PS	10	0.37	0.46	0.04	7.03	3.31
Soil + SRB + PS	10	0.53	0.52	0.04	7.61	4.02

**Figure 4:**
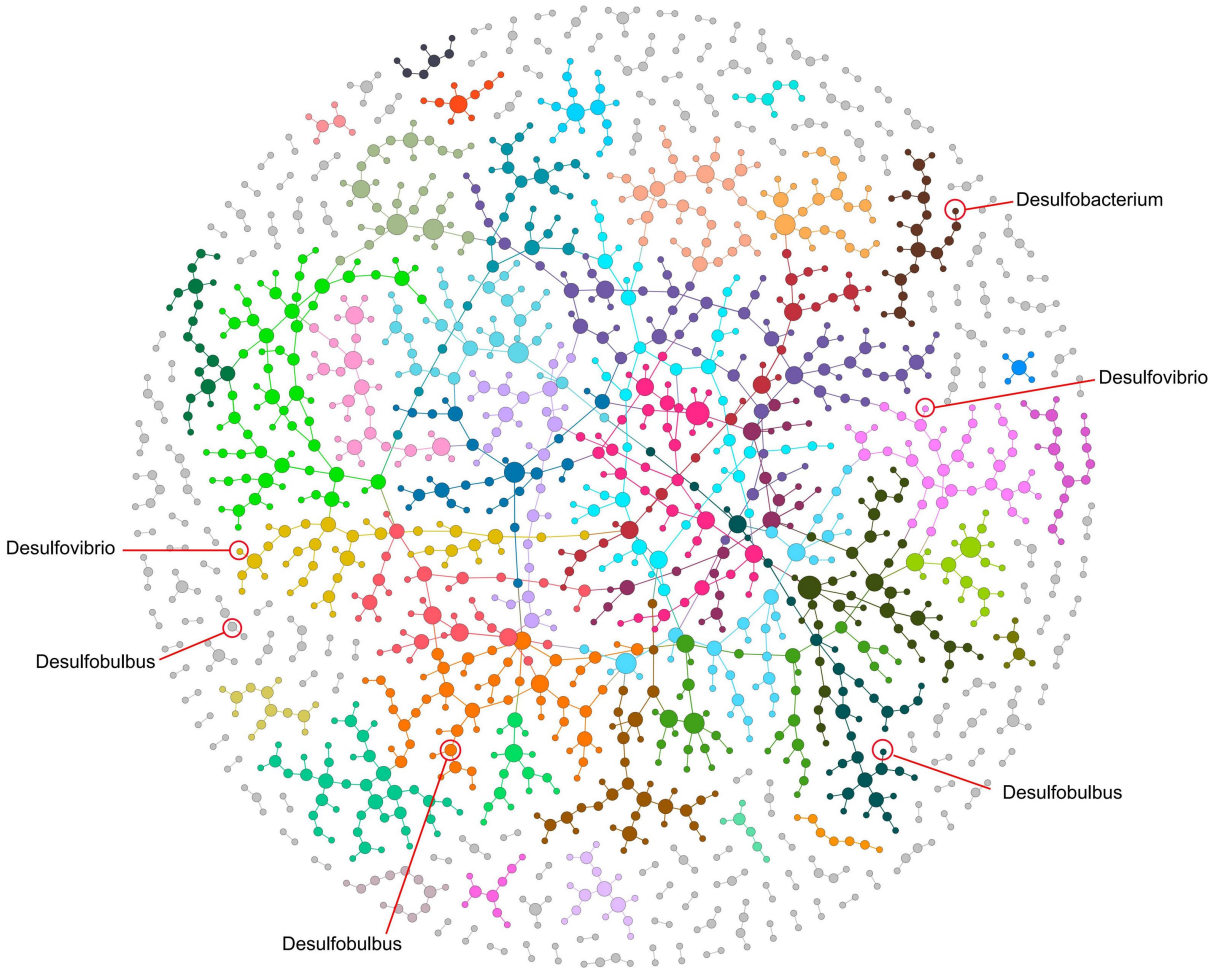
Ecological network at a global scale encompassing all setups, illustrating correlations among microbes. Each node represents an OTU, and links between nodes indicate a significant Pearson’s correlation (*p* < 0.05) in their relative abundances. Genera lacking correlations with others have been filtered out. Modules, delineated by distinct colors, were identified using the fast-greedy method. Three genera of SRB, including *Desulfomicrobium*, *Desulfovibrio*, and *Desulfobulbus*, are integrated within these modules, playing crucial roles in network connections.

To further reveal the synergies and rivalries among SRB and their microbial counterparts in coal mine-impacted soil, we embarks on crafting a holistic ecological interaction network spanning all treatment conditions (Figure 4). At a panoramic view, this network unfurls into a meticulously ordered modular construct, encompassing a staggering 198 modules. This mirrors a labyrinth of microbial communications and specialized factions. Within this expansive microbial mosaic, pivotal SRB genera, including *Desulfovibrio*, *Desulfobulbus*, and *Desulfobacterium*, carve their niche. *Desulfovibrio*, marking its presence in Modules M9 and M8, emerges as a potential linchpin, potentially orchestrating myriad functional microbial alliances. Meanwhile, *Desulfobulbus* weaves its narrative across Modules M60, M10, and M3, signaling its multifaceted engagements in the original SRB consortium. *Desulfobacterium*, predominantly stationed in M25, adds another layer of complexity. Notably, the *Desulfobacter*, initially identified in the inoculum, conspicuously fades from the network, suggesting ecological displacement and potential competitive exclusion during the soil restoration trajectory.

Zooming into the finer interaction matrices, ecological role of *Desulfovibrio* reveals a positive rapport with a spectrum of genera, including the likes of *Limnobacter*, *Sandaracinus*, and *Cellulomonas*, indicating probable cooperative endeavors. In contrast, its subtle skirmishes with genera like *Anaeromyxobacter* and *Rhodocytophaga* which might reflect ecological contests, potentially rooted in resource skirmishes overlaps (Albornoz et al., 2022). The interaction tableau of *Desulfomicrobium* presents its own set of fascinations. It forges positive ties with genera such as *Ornithinibacter* and *Rhodocytophaga*, while navigating contentious terrains with *Bacillus* and its ilk. These dynamic interplays possibly echo the metabolic predilections and ecological imperatives of these microorganisms within the coal mine ecosystem, spotlighting their coexistence in diverse habitats. On the other hand, with *Desulfovibrio* championing sulfate reduction and *Sphingomonas* acclaimed for phosphorus solubilization, their interplay underscores a likely mutualistic synergy. Together, these results suggest that the introduction of SRB significantly influenced the microbial network in the soil, establishing linkages that unify the modified ecological functions.

### 3.6. Metabolic profiling of microbial functional and enzymatic responses across treatments

**Figure 5:**
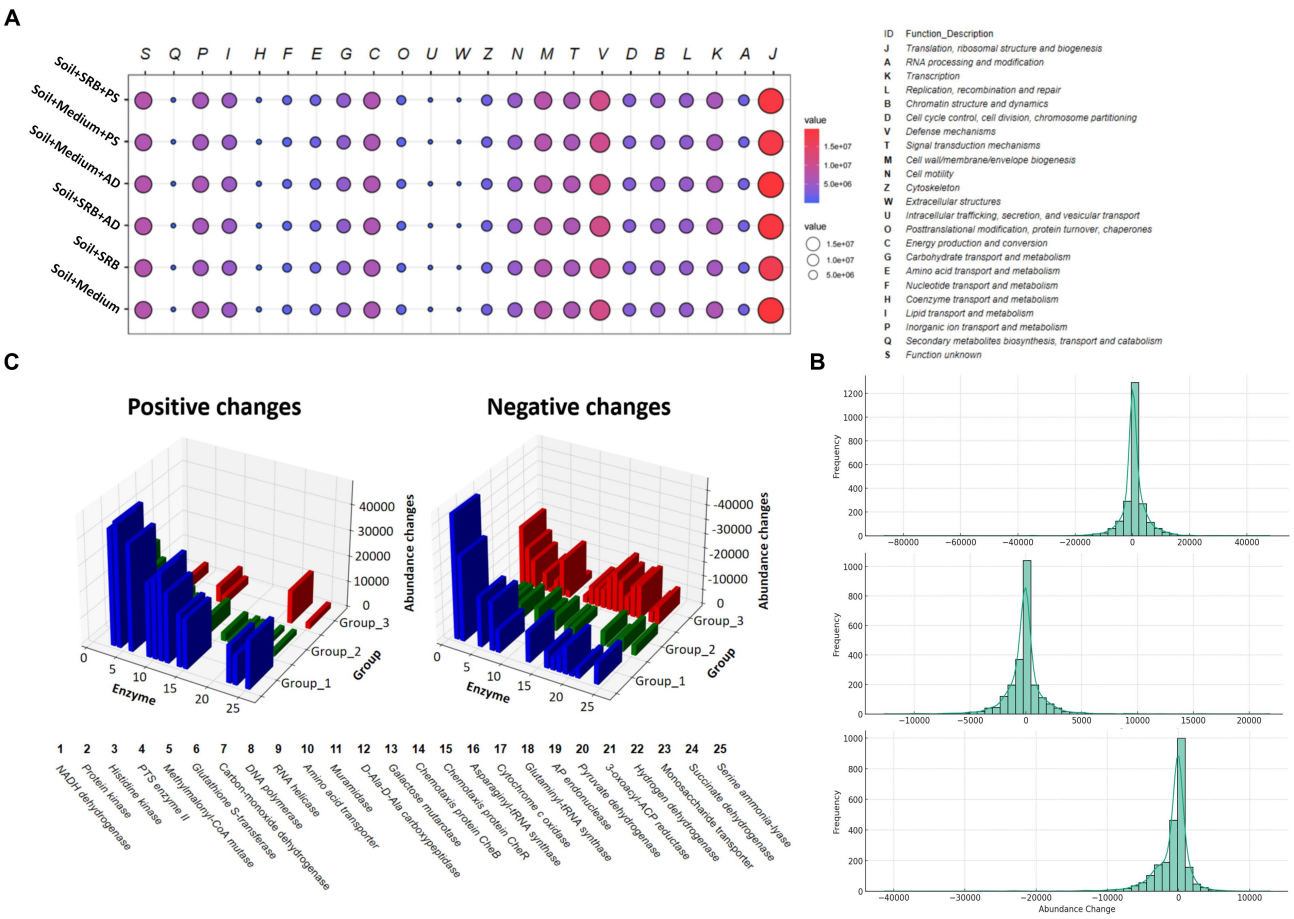
Functional categorizations and changes in enzyme abundance across various setups. **(A)** Bubble plot illustrating the functional categorizations at level 1, showcasing different biomolecular processes along with their corresponding values. **(B)** Histograms presenting the distribution of enzyme abundance changes in three distinct groups. The Group 1 is displayed at the top; Group 2 is displayed in the middle; Group 3 is displayed at the bottom. Each histogram is complemented with a Kernel Density Estimation (KDE) overlay, offering a smoothed representation of the distribution to facilitate a more intuitive understanding of the data trends. **(C)** 3D diagram highlighting the abundance changes of the top 25 most affected enzymes when SRB is added, in comparison to setups without SRB. This comparative analysis provides a visual representation of the substantial impact SRB has on enzyme abundance in each group.

Metabolic prediction is conducted to decipher microbial functional changes across various soil treatments. Figure 5A shows microbial functional abundance across varied soil treatments, distinct patterns emerge, elucidating their adaptive responses to specific cues. The SRB supplemented soil exhibits notable increases in functions related to *Inorganic Ion Tran*spor*t and Metabolism and Transcription*. These variations are indicative of the inherent metabolic pathways of SRB. Actively engaged in the sulfur cycle, SRB convert sulfate to sulfide, necessitating enhanced inorganic ion transport and metabolic activity (Dong et al., 2019). The heightened transcriptional activity underscores active metabolic state of SRB, suggesting an upregulation of genes central to sulfate metabolism (Yu et al., 2022). In the AD-integrated soil, both with SRB and solely with the medium, there is an evident augmentation in *Amino Acid Transport and Metabolism* and a marked increase in *Carbohydrate Transport and Metabolism*. This pattern hints at significant impact of AD on the microbial milieu. The potential release of carbohydrate-laden root exudates from AD could provide substrates for microbial carbohydrate metabolism (Adamczyk et al., 2021). Additionally, these exudates might stimulate microbial communities to intensify amino acid transport and metabolism, mirroring established plant-microbe interactions (Vurukonda et al., 2022). Contrastingly, soil integrated with PS displayed a subtle decline in amino acid-related functions compared to their counterparts without PS.

Figure 5B elucidates the enzyme abundance changes across different treatments, underscoring the subtle intricacies of microbial enzymatic adaptations. The histogram reveals that the SRB inoculated soil, compared to its medium-only counterpart, exhibits a unique bimodal distribution. This dual-peaked pattern suggests two primary groups of enzymes reacting differently, potentially due to varied influences on biological pathways. On the other hand, the soil supplemented with SRB and AD, when juxtaposed against the AD and medium control, predominantly showcases stability in enzyme abundance. This indicates that the presence of AD might attenuate some of the pronounced effects seen with SRB alone. Conversely, the combination of SRB and PS, relative to its control, demonstrates only slight alterations in enzyme abundance, emphasizing a more balanced microbial response. Across all treatments, there is a noticeable trend of both enzyme upregulation and downregulation. This duality reflects the complex interplay of microbial communities reacting to their environments. Notably, certain enzymes in the SRB inoculated soil experience significant shifts in abundance. These marked changes indicate these enzymes’ heightened responsiveness to the treatment, shedding light on potential critical metabolic pathways influenced by SRB in the soil matrix.

Further, identifying these enzymes and understanding their roles can illuminate the metabolic pathways primarily influenced by different treatments, offering insights into microbial metabolic shifts in these soil. Figure 5C, detailing the top 25 affected enzymes, provides a glimpse into enzymatic adaptations across different soil treatments. In soil inoculated with SRB compared to those with only the medium, there is a significant shift in *NADH:ubiquinone reductase* [*H*(*+*)*-translocating*], signaling alterations in microbial energy metabolism (Vitt et al., 2022). This aligns with the known role of SRB in sulfate reduction. The increased activity of *Non-specific serine/threonine protein kinase* further highlights the resilience and adaptability of the microbial community. The presence of enzymes such as *Carbon-monoxide dehydrogenase* (*acceptor*) and *UDP-glucose 4-epimerase* indicates potential changes in how microbes metabolize carbon, which can directly influence metal binding and mobility within soil (Wiatrowska and Komisarek, 2019). For soil supplemented with both SRB and AD, the prominence of *DNA-directed DNA polymerase* suggests an active DNA repair mechanism (Hamilton et al., 2016), vital for mitigating potential metal-induced stresses. Concurrently, enzymes like *Cytochrome-c oxidase* and *Hydrogen dehydrogenase* [*NADP*(*+*)] may facilitate electron transfer processes that contribute to metal reduction and immobilization (Joseph et al., 2021), especially in the presence of organic exudates from AD. Conversely, in soil combined with SRB and PS, the enzyme profile emphasizes the influence of PS on microbial nutrient dynamics. These enzymatic shifts emphasize the foundational role of SRB, synergized by plants like AD and PS, in driving microbial strategies that undergird metal sequestration and holistic soil restoration.

## 4. Conclusion

To conclude, this study collectively presents substantial evidence underlining the critical role of sulfate-reducing bacteria (SRB) in bioremediation of heavy metal contamination induced by coal mining. The results demonstrate that introducing specialized SRB consortium significantly improves metal sequestration potential, thereby decreasing the environmental impact. Furthermore, the plants *Acacia dealbata* and *Pisum sativum* co-exist productively with SRB, enhancing microbial diversity which subsequently improves soil restoration effectiveness. The synergies in this assemblage strengthen ecological relationships, enhancing microbial resistance and promoting an active network bolstered by vital survival functions. Our findings underscore the potential of SRB-focused bioremediation, combined with specific plant growth, as a viable approach for rehabilitating heavy-metal contaminated soils, especially in regions, such as Midwest China, affected by coal mining activities. Yet, the complex nature of plant-root exudates and their impact on these synergies requires further research to optimize the efficacy of these bioremediation strategies.

## Data availability statement

The data presented in this article can be accessed by accession PRJNA1034992 and at this https://www.ncbi.nlm.nih.gov/bioproject/PRJNA1034992.

## Author contributions

ZheY: Conceptualization, Data curation, Formal analysis, Investigation, Methodology, Resources, Validation, Visualization, Writing – original draft. QW: Funding acquisition, Project administration, Supervision, Writing – review & editing. ZL: Data curation, Formal analysis, Writing – original draft. XQ: Investigation, Validation, Writing – review & editing. ZZ: Software, Visualization, Writing – review & editing. MH: Writing – review & editing, Data curation, Resources. CP: Writing – review & editing, Investigation, Validation. LZ: Writing – review & editing, Methodology, Software. JW: Writing – review & editing, Visualization. FL: Writing – review & editing, Funding acquisition, Project administration, Supervision. ZhaY: Writing – review & editing, Data curation, Resources. HY: Writing – review & editing.
